# Large –scale wheat flour folic acid fortification program increases plasma folate levels among women of reproductive age in urban Tanzania

**DOI:** 10.1371/journal.pone.0182099

**Published:** 2017-08-10

**Authors:** Ramadhani A. Noor, Ajibola I. Abioye, Nzovu Ulenga, Salum Msham, George Kaishozi, Nilupa S Gunaratna, Ramadhani Mwiru, Erin Smith, Christina Nyhus Dhillon, Donna Spiegelman, Wafaie Fawzi

**Affiliations:** 1 Africa Academy for Public Health (AAPH), Dar es Salaam, Tanzania; 2 Department of Global Health and Population, Harvard T. H. Chan School of Public Health, Boston, Massachusetts, United States of America; 3 Department of Nutrition, Harvard T. H. Chan School of Public Health, Boston, Massachusetts, United States of America; 4 Management Development for Health (MDH), Dar es Salaam, Tanzania; 5 Helen Keller International, Dar es Salaam, Tanzania; 6 Department of Epidemiology, Harvard T. H. Chan School of Public Health, Boston, Massachusetts, United States of America; 7 Department of Biostatistics, Harvard T. H. Chan School of Public Health, Boston, Massachusetts, United States of America; Universidade de Sao Paulo, BRAZIL

## Abstract

There is widespread vitamin and mineral deficiency problem in Tanzania with known deficiencies of at least vitamin A, iron, folate and zinc, resulting in lasting negative consequences especially on maternal health, cognitive development and thus the nation’s economic potential. Folate deficiency is associated with significant adverse health effects among women of reproductive age, including a higher risk of neural tube defects. Several countries, including Tanzania, have implemented mandatory fortification of wheat and maize flour but evidence on the effectiveness of these programs in developing countries remains limited. We evaluated the effectiveness of Tanzania’s food fortification program by examining folate levels for women of reproductive age, 18–49 years. A prospective cohort study with 600 non-pregnant women enrolled concurrent with the initiation of food fortification and followed up for 1 year thereafter. Blood samples, dietary intake and fortified foods consumption data were collected at baseline, and at 6 and 12 months. Plasma folate levels were determined using a competitive assay with folate binding protein. Using univariate and multivariate linear regression, we compared the change in plasma folate levels at six and twelve months of the program from baseline. We also assessed the relative risk of folate deficiency during follow-up using log-binomial regression. The mean (±SE) pre–fortification plasma folate level for the women was 5.44-ng/ml (±2.30) at baseline. These levels improved significantly at six months [difference: 4.57ng/ml (±2.89)] and 12 months [difference: 4.27ng/ml (±4.18)]. Based on plasma folate cut-off level of 4 ng/ml, the prevalence of folate deficiency was 26.9% at baseline, and 5% at twelve months. One ng/ml increase in plasma folate from baseline was associated with a 25% decreased risk of folate deficiency at 12 months [(RR = 0.75; 95% CI = 0.67–0.85, P<0.001]. In a setting where folate deficiency is high, food fortification program with folic acid resulted in significant improvements in folate status among women of reproductive age.

## Introduction

Multiple micronutrients deficiencies including iron, folate and vitamin A are key contributors to morbidity and mortality globally[1]. In Tanzania, anemia affects 40% of women of reproductive age, with deficiencies in iron and vitamin A measuring 30% and 36%, respectively[2]. Maternal folate insufficiency has serious consequences to newborns, and among them is the neural tube defects (NTDs)[3–5]. In Tanzania like most developing countries, limited estimates exist on the magnitude of folate deficiency[2,6,7]. As a proxy measure and sequel of folate deficiency among women of reproductive age, the birth prevalence of neural tube defects (NTDs) in Tanzania is estimated to be as high as 3 NTDs per 1000 live births[8,9]. It is estimated that micronutrient deficiencies cost Tanzania over US$ 518 million, estimated at 2.65% of the country’s GDP annually[10]. Beyond the economic losses, vitamin and mineral deficiencies are a significant contributor to infant mortality, with over 27,000 infant and 1,600 maternal deaths annually attributable to this cause[10].

Adequate consumption of folic acid before pregnancy and during the early weeks of gestation decreases the risk of developing NTDs[11–14].Hence, there is a global recommendation for peri-conceptional supplementation with 400 micrograms per day (μg/d) of synthetic folic acid for women of child-bearing age beginning at least 1 month before conception through the first 3 months of pregnancy[15,16]. High rates of unplanned pregnancies, poor adherence as well as late reporting to ante natal care severely undermine the success of supplementation programs[1,6,17,18], and the impact of continuing with the peri-conceptional supplementation after the first trimester of pregnancy remains unclear[19].

Food fortification is described as the single most cost-effective public health strategy for preventing and controlling micronutrient deficiencies[20–22]. The first cereal grain fortification recommendations issued by the World Health Organization (WHO) was for wheat flour and maize flour[23]. And as of 2015, 83 countries have mandated wheat flour fortification with iron and/or folic acid; and 16 countries have mandated maize flour fortification with the same nutrients [24]. Evidence on the public health impact of these programs suggests folic acid fortification of flour is effective in reducing neonatal mortality and NTDs[25], however the evidence from developing countries, particularly in Africa is limited[26,27]. This evaluation was therefore designed first, to determine the prevalence of folate deficiency in this cohort of women of reproductive age prior to a food fortification program rollout and hence validate potential for benefit in Tanzania, and second, to assess the effectiveness of the national food fortification program by examining prospective folate levels as a proxy measure for public health impact in Tanzania.

## Materials and methods

### Food fortification program

The Tanzania National Food Fortification Program mandates fortification[28] to targeted staple foods including salt, edible oil, wheat and maize flour Table 1. With the exception of salt, which has been fortified with iodine since 1994, fortification of other food staples officially started in 2013 through a mandate passed by the government of Tanzania requiring all industrial processed wheat, maize and edible oils to be fortified. This study is limited to wheat flour fortification only since folic acid fortification of maize flour had not been implemented to scale at the time of the study.

**Table 1:** Fortification of staple foods in Tanzania.

Staple	Year started	Nutrient	Fortificant compound	mg/kg
Wheat	2013	Iron	Sodium Iron EDTA	40±10
		Zinc	Zinc Oxide	40±10
		Vitamin B12	Vitamin B12 0.1% WS^a^	0.015±0.005
		Folate	Folic acid	3±2
Maize	2013	Iron	Sodium Iron EDTA	10±5
		Zinc	Zinc Oxide	30±10
		Vitamin B12	Vitamin B12 0.1% WS^a^	0.005±.002
		Folate	Folic acid	1.5±1
Cooking Oil	2013	Vitamin A	Retinyl Palmitate	28

^a^WS = Water-Soluble

Helen Keller International worked in partnership with the Ministry of Health and Social Welfare through Tanzania Food and Drug Authority (TFDA), the Tanzania Food and Nutrition Centre (TFNC), the Ministry of Industry and Trade, the Tanzania Bureau of Standards (TBS), and food producers to assist in the rollout of this mandate. To date 14 large-scale food producers participate in the national program, including all ten of the country’s wheat flour producers, and four large-scale vegetable oil refineries. Production capacity for fortified products among these 14 industries amounts to approximately one million metric tons (MT) of wheat flour and 300,000MT of vegetable oil annually, or roughly 88% and 80% of the market share, respectively. TFDA works to ensure fortified products meet the levels of quality required by standards set by Tanzania Bureau of Standards (TBS) at production point, in the market, and at ports of entry through regular inspections.

**Fig 1:**
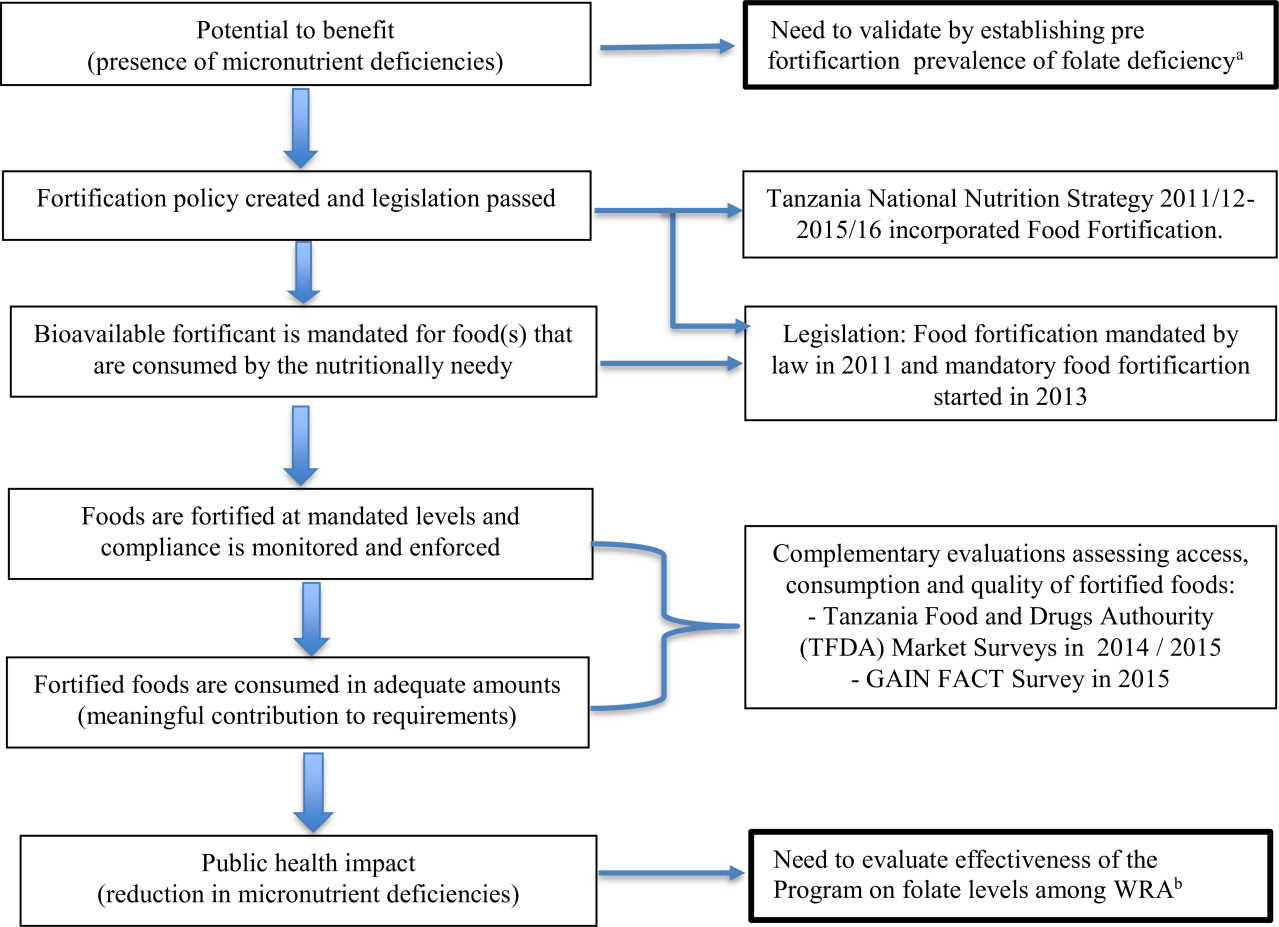
Program Impact Pathway (PIP) for large-scale food fortification programs. Adopted with modifications from Reynaldo Martorell et al. Am J Clin Nutr 2015; 101:210–217. ^a,b^Areas of focus for this evaluation in the context of Tanzania National Food Fortification program.

Using the program impact pathway adopted from a similar evaluation undertaken in Costa Rica S1 File.[29], we mapped an impact pathway for the Tanzania Food Fortification program and identified the scope and key areas of focus for this evaluation (Fig 1).

### Study design, settings and participants recruitment

We conducted a prospective cohort study comparing participant’s plasma folate levels before and after the rollout and scale up of the national food fortification program. We enrolled non-pregnant women of reproductive age (18–49 years) living in the Temeke and Ilala districts of Dar es Salaam. These two districts were selected to provide a mix of urban and peri-urban populations assuming these communities would benefit from relatively faster access to fortified foods, given their close proximity to the main food industries in Dar es Salaam. To maintain maximum representation with high internal and external validity, participants were recruited from 10 clinics, 5 from each of the two selected districts, providing a 90% aggregate coverage of antenatal care for pregnant women within the population catchment area. Given the high rates of antenatal clinic (ANC) coverage by these clinics, we assumed that eligible participants attending Mother and Child Health (MCH) clinics from these facilities are a representative sample for women of reproductive age in the two districts selected for this study.

One district hospital and four health centers MCH clinics were selected from each, Temeke and Ilala districts. We invited women attending clinics with their children for immunization program appointments to participate in this study. We randomly selected women to participate through a lottery system using identical folded cards with “YES” and “NO” labels. Women who picked “YES” were further screened for the study. An equal number of participants were enrolled across the ten participating sites giving a total of 60 participants from each site.

### Sample size

Since no pre-existing data on prevalence of folate deficiency and effect size from the region could be found for reference, our sample size was calculated using estimates obtained from similarly designed studies in Latin America[30]. Assuming a two-sided alpha of 0.05, a power of 0.8, intraclass correlation of 0.02, and mean plasma folate levels of 10±7 and 13±9 ng/ml at baseline and end point, respectively, we obtained a sample size of 416 participants. In order to account for loss to follow up in this study, we inflated the sample size by roughly 50%, resulting in a total sample of 600 women of reproductive age.

### Enrolment and follow up

**Fig 2:**
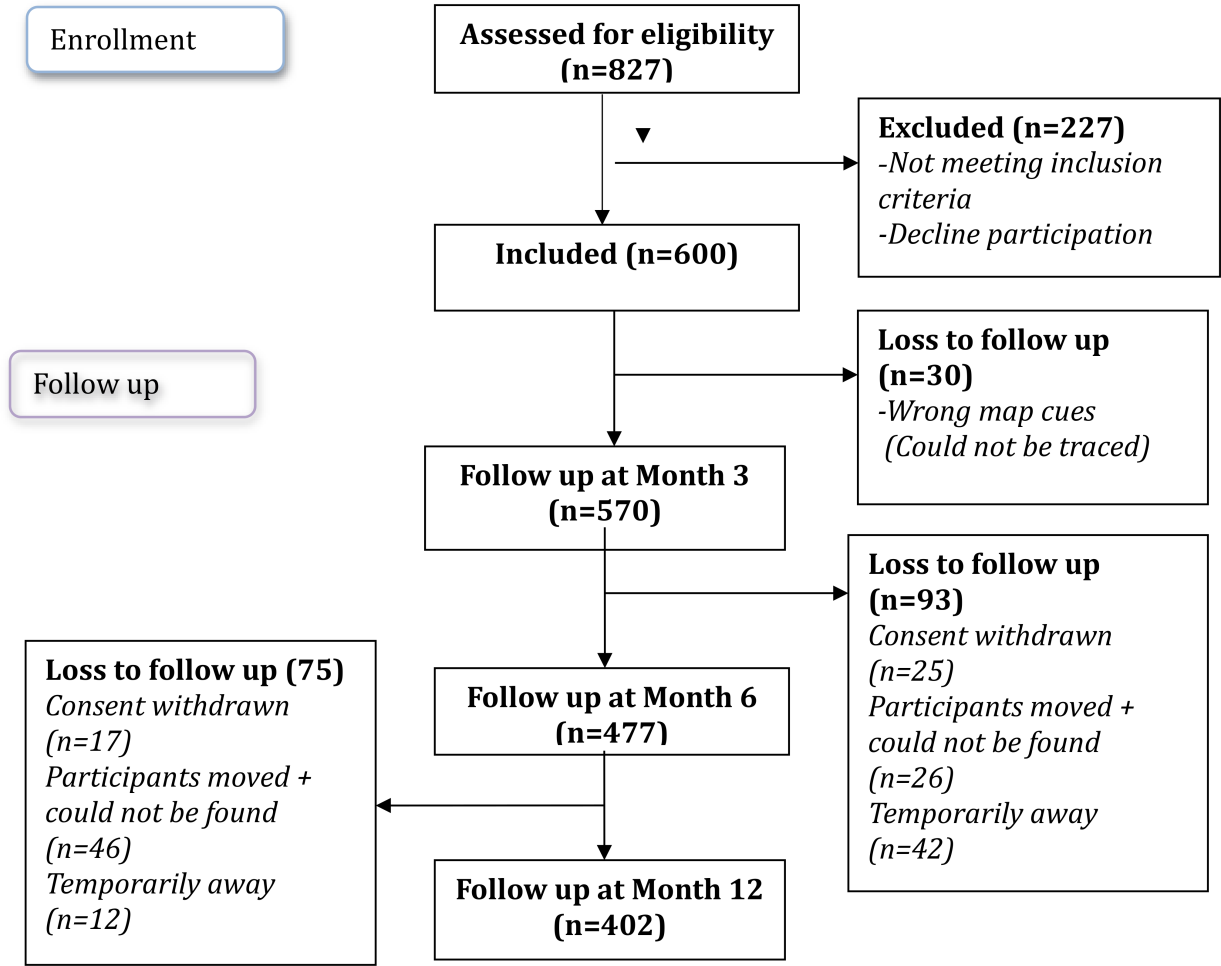
Study flow diagram on the numbers of participants enrolled and followed up during the study period.

We enrolled 600 out of 827 women screened across the 10 study sites in Dar es Salaam (Fig 1). Inclusion criteria was based on participants not being pregnant, based on the date of their last menstrual period as well as a urinary test during screening, having given birth at least 9 months prior to study inclusion, planning to remain in the study area for the next 12 months, and giving written informed consent to participate in the study. We excluded participants who were currently or had previously taken folic acid/iron supplements during the past 9 months, and anyone with a reported chronic illness (Fig 2).

The plasma folate status of enrolled participants was assessed at baseline, and at 6 and 12 months. During these visits, fasting blood samples of ten hours or greater were obtained to determine the plasma folate levels. To maintain contact with our participants and to enhance follow-ups, we conducted home visits and phone calls to all participants during months where no clinic visits were scheduled to provide nutrition counselling on nutrient rich diverse diets, use of fortified foods water and sanitation practices. We also collected dietary intake data from those who have missed their clinic visits as well as reminding participants on their upcoming clinic visit.

### Ethical consideration

Written, informed consent for voluntary participation was obtained from each study participant before enrolment. The consent process was conducted in Kiswahili, the local language. The research protocol and the consent forms were reviewed and approved by the Medical Research Review Council of the National Institute for Medical Research (MRRC–NIMR), Tanzania and the Institutional Review Board of the Harvard T. H. Chan School of Public Health, USA. In accordance to Tanzania standard of care, nutritional counselling was provided to all participants.

### Measurement of dietary intake

A dietary intake assessment using a semi-quantitative FFQ, validated for this setting was conducted at baseline, and at 6 months, and 12 months of follow up to allow estimation of total intake as well as isocaloric comparison and interpretation of the plasma folate results [31]. The questionnaire was comprised of 108 food items commonly consumed. Participants were asked if they had consumed these foods in the prior month, and if so, how often, and the frequencies were converted to servings per day. A serving is based on food specific portion sizes, using the Tanzania Food Composition Tables[32]. Using this table, we computed the food folate, which is comparable to the dietary folate equivalents (DFE)[33] as well as total energy consumed. We restricted data to FFQs with reasonable total energy intake, >600kcals and <4500kcals.

### Specimen collection and quality control

Venous blood specimens (about 4 ml) were collected in purple top tubes containing K_2_EDTA using standard venepuncture procedures, and samples were sent to the Africa Academy of Public Health (AAPH) laboratory within two hours of collection. Once in the laboratory, specimens were centrifuged at 3200 rpm for ten minutes to obtain plasma that was stored in a -20C freezer in 1 to 3ml vials. Laboratory scientists made sure that specimens were free from hemolysis, lipemia and icterus.

Plasma folate assays were done using the Cobas e411 automated analyzer (Roche Diagnostics, Switzerland) as per manufacturer instructions. The machine was calibrated by lyophilized human plasma with folate. Adding 1.0 ml of distilled water carefully dissolved the contents of the calibrators. The calibrators were mixed carefully, avoiding foam formation. Aliquots of the reconstituted calibrators were transferred into empty vials and stored at -20°C till needed. Whenever required, a pair of calibrators were brought out and thawed before use at room temperature. Testing proceeded after successful calibration, with 250–300μl of each sample run in duplicate and average readings recorded. Negative and positive controls were used for quality assurance in each run. Controls were run individually at least once per 24 hours while the test was in use, once per new reagent kit and after each passed calibration. Printed results were certified by one laboratory personnel and reviewed by another one, before entered into a database. The normal range for the equipment is 1.5–20.0 ng/ml.

### Statistical analysis

We analysed change in mean plasma folate levels from baseline, to 6, and 12 months of follow-up. Mean (±SD) values of total energy intake (in calories/day), macronutrient proportions of the total calorie intake, as well as intake of fortified wheat-based foods (in servings/day) were calculated to estimate folic acid intake at baseline. We estimated the change in levels of these measures from baseline, and assessed statistical significance using a paired Student’s T-test.

The concentrations suggested for defining folate deficiency based metabolic indicators range from 3–4 ng/ml [15,16,34]. This value is derived from data related to preventing anaemia and hyperhomocysteneinemia and the public significance of applying same cut-off in isolating folate deficiency in the context of NTDs is not fully understood[15,34]. Evidence suggests risk of NTD increases with folate insufficiency levels higher than ranges defining folate deficiency[34]. We dichotomized plasma folate levels based on the stringent threshold indicative of folate deficiency using a cut-off point of 4 ng/ml[15,16]. We fit univariate and multivariate binomial regression models to assess the degree to which each unit change in plasma folate led to a change in the risk of folate deficiency at six and twelve months, and obtained risk ratio estimates[35]. In multivariate models, we included potential confounders known to be associated with folic acid intake and/or plasma folate levels, or have been identified in regression models (p<0.2) to be significantly related to dietary intake of folic acid at baseline. Relative risks were adjusted for age (18 to <2 6, 26 to <36 and 36–49 years), years of formal education (0–7 years, 8–11 years and ≥12 years), occupation (business/professional, skilled formal, skilled informal, unskilled, unemployed), body mass index (<18.5, 18.5 to <25, 25 to <30, ≥30 kg/m^2^), household dietary diversity score (1–12), baseline intake of fortified wheat-based foods (servings per day), intake of vegetables and total energy intake (kcals/day).The number of household assets were computed from a simple count that included TV, radio, generator, fan, bike, car, couch, fridge, as well as access to electricity and potable water, allowing classification into 3 socioeconomic groups thus: 0–5, 6–8, and 9–10 [36]. Covariates with missing data were retained in the analysis using the missing indicator method[37].P-values were two-sided and significance was set at < 0.05. Final data set S2 File was compiled and all statistical analyses were conducted using SAS version 9.2 (SAS Institute Inc).

## Results

A total of 600 non-pregnant women of reproductive age were enrolled and followed for a period of 12 months Table 2. The mean age (±SD) of the participants was 28 years (±7). A majority of the participants had completed seven years of formal education or less (68%) and were unemployed (51%). All the participants were found to be in the low socioeconomic class. The mean (±SD) body mass index (BMI) was 24.4 (±5.0) kg/m2. Mean dietary folic acid intake was low (<500μg/d) at baseline in 66% of the participants.

**Table 2:** Basic Characteristics of women at baseline (n = 600).

	Categories	Percent
Age (Years)	Mean (±SD)	28.4 (±6.7)
	18–<26	39%
	26–<36	43.5%
	36+	17.5%
Years of completed education	0–7	68.3%
	8–11	23.7%
	12+	8%
Occupation	Unemployed	51.0%
	Unskilled	29.5%
	Skilled informal	0%
	Skilled formal	3.0%
	Business/professional	16.5%
Total household assets	0–5	22.3%
	6–8	53.7%
	9–10	24%
Family expenditure on food	Mean (±SD)	7,960(±4465)
(Per Day)	<10,000 TZS^a^	66.5%
	≥10,000 TZS^a^	33.5%
Where family buys groceries	Local retail shops	93.5%
	Others incl. supermarkets	6.5%
Body mass index (kg/m sq)	Mean (±SD)	24.4 (±5.0)
	<18.5	7.5%
	18.5 to <25	58.7%
	25 to <30	21.3%
	30+	12.5%
Dietary folic acid intake	Mean (±SD)	456 (±187)
	Low (<500μg/d)	66.2%
	Adequate	33.8%

^a^10,000 Tanzania Shillings which is approximately equal to 4 USD

At baseline, the mean (±SD) energy intake based on intake surveys was 2906 kcals/day (±966). There was significant reduction in the total energy intake among participants during follow-up, with an average drop of 599 kcals (P<0.0001) and 700 kcals (p<0.0001), compared to baseline at month 6 and 12 respectively. Energy intake was comprised of protein (12%), fat (32%) and carbohydrate (56%) on average. There was a slight increase in protein intake (0.52%; p-value = 0.006. Besides this, dietary composition was materially unchanged over the course of follow up, with no significant change in carbohydrate as well as fat intake at any time point. The mean (± SE) serving per day of fortified wheat-based foods at baseline was estimated to be 0.84 (±0.56). There was significant reduction in wheat-based foods intake (-0.23 servings/day) and (-0.25 servings per day), compared to baseline intake by the sixth and twelve month of follow-up respectively Table 3.

**Table 3:** Macronutrient and fortified wheat flour intake among non-pregnant women of reproductive age in Dar es Salaam, Tanzania.

	Baseline (n = 537)	Six months (n = 413)		12 months (n = 361)	
Measure	Intake (mean*±SD)*	Intake(mean*±SD)*	Change; p-value	Intake (mean*±SD)*	Change; p-value
Energy (kcals/day)	2906 (±966)	2447 (±851)	-599; <0.0001	2348 (±860)	-700; <0.0001
Protein %	12.2% (±2.5)	12.7 (±2.57)	0.52; 0.006	12.5 (±2.76)	0.30; 0.17
Fat %	31.8% (±6.27)	31.7 (±5.95)	0.45; 0.29	32.3 (±5.81)	0.88; 0.08
Carbohydrate %	56.0% (±7.9)	55.5 (±7.36)	-0.97; 0.06	55.1 (±7.39)	-1.18; 0.07
Fortified wheat-based foods^a^ *(meanservings/day±SE)*	0.84 (±0.56)	0.61 (±0.28)	-0.23; <0.0001	0.62 (±0.55)	-0.25; <0.0001

^a^Wheat-based foods included bread, pancakes, cakes and donuts

N’s are different at each time point due to FFQ inclusions for analysis

The mean plasma folate concentrations were 5.44 ng/ml (±2.30) at baseline, 10.08ng/ml (±2.57) at six months and 9.70ng/ml (±3.75) at twelve months Table 4. There was an increase of mean plasma folate of 4.57ng/ml (±2.89) from baseline to 6 months and 4.27 ng/ml (±4.18) from baseline to 12 months. The slight drop in folate levels from six to twelve months was not significant (p = 0.27). We compared baseline values of all variables in those who completed the study versus those lost to follow-up, and we found no differences in those two groups (p>0.05). Overall, there were significant reductions in the risk of folate deficiency at six and twelve months of follow-up Table 4. There was a 25% (15%–32%) reduction in the risk of folate deficiency at 12 months for every 1ng/ml increase in plasma folate from baseline levels.

**Table 4:** Risk of folate deficiency among non-pregnant women of reproductive age in Dar es Salaam, Tanzania.

Measures	Baseline (n = 511)	Six months (n = 410)	p-value	12 months (n = 366)	p-value
Mean (±SD)	5.44 (±2.30)	10.08 (±2.57)		9.70 (±3.75)	
Difference (±SD)		4.57 (±2.89)	<0.0001	4.27 (±4.18)	<0.0001
% Deficient	26.9%	0.5%		5.0%	
For every 1ng/ml increase in plasma folate					
Univariate RR (95% CI) ^a^	1.00	0.76 (0.49, 1.18)	0.23	0.74 (0.68, 0.81)	<0.0001
Multivariate RR (95% CI) ^a^^,^^b^	1.00	0.78 (0.51, 1.20)	0.26	0.75 (0.67, 0.85)	<0.001
Folate deficiency at baseline^c^					
Univariate RR (95% CI) ^a^	1.00			0.60 (0.17, 2.08)	0.42
Multivariate RR (95% CI) ^a^^,^^b^	1.00			0.47 (0.10, 2.11)	0.32
On restriction to participants with complete follow-up^d^					
For every 1ng/ml increase in plasma folate					
Univariate RR (95% CI) ^a^	1.00			0.75 (0.67, 0.82)	<0.0001
Multivariate RR (95% CI) ^a^^,^^b^	1.00			0.74 (0.64, 0.86)	<0.0001
Folate deficiency at baseline, *complete follow-up*^d^					
Univariate RR (95% CI) ^a^	1.00			0.63 (0.14, 2.90)	0.55
Multivariate RR (95% CI) ^a^^,^^b^	1.00			0.82 (0.16, 4.10)	0.81

^a^Relative risk (RR) estimates were obtained from binomial regression models. RR above 1 suggests an increased risk of folate deficiency for every one-unit increase in the plasma folate. RR below 1 suggests a decreased risk of folate deficiency for every one-unit increase in the plasma folate.

^b^Multivariate estimates were adjusted for age in years (15–<26, 26–<36, ≥36), educational status in completed years (0–7, 8–11, ≥12), occupation (unemployed, unskilled, skilled informal, skilled formal, business/professional), total assets (0–5, 6–8, 9–10), minimum dietary diversity score for women (>5, ≤5), BMI, and the intake of wheat-based foods (<1 serving/day, ≥1 serving/day), vegetables (<1 serving/day, ≥1 serving/day) and total energy (kcal) at baseline

^c^Folate deficiency was defined as plasma folate concentration <4ng/ml

^d^Restricted to 298 participants with follow-up at six and twelve months

## Discussion

Tanzania provides a model for mandatory large-scale food fortification in Africa[38]. Our evaluation on the effectiveness of this program demonstrates 6 months after the introduction of the program, a significant reduction in the prevalence of folate deficiency occurred in a cohort of women of reproductive age, with the benefit persisting up to 1 year after the program roll-out. This evaluation provides one of the first results on effectiveness of food fortification programs in Africa, in a setting where the prevalence of folate deficiency among women of reproductive age was high. Our results indicate that folate insufficiency remains prevalent in this region despite existing dietary as well as peri-conceptional folic acid supplementation programs. Hence, there is an important role for fortification programs as a cost effective large-scale intervention to address folate and other micronutrient deficiencies [20–22]. There is strong evidence that folate insufficiency is significantly associated with higher risk of NTDs[34] and increased neonatal mortality, as well as a contributor to anaemia. Hence, preventing folate deficiency in women of reproductive age is likely to significantly reduce child mortality[3].

To our knowledge, this is the first evaluation of folic acid intake among non-pregnant women of reproductive age using both dietary intake information and biochemical markers of folate status in sub Saharan Africa. On average, women of reproductive age in this study, consumed diets containing adequate energy and macronutrient distribution in compliance with international recommendations for healthy eating[39]. At baseline, the energy intake was comprised of protein (12%), fat (32%) and carbohydrate (56%). The mean intake values for fat and carbohydrates did not differ markedly from the fat and carbohydrate intakes observed in other African populations[40]. However, the vast majority of women in this study did not meet the recommended intakes for folic acid intake before fortification. Similar inadequate intakes of folic acid have also been reported among Tanzanian rural and urban children as well as among the elderly population [41].

The mean serving per day of fortified wheat-based foods at baseline was estimated at 0.84, confirming that wheat flour is an appropriate vehicle for fortification in this population. This has direct implications for fortification programs in sub-Saharan Africa, where cereals, including wheat flour contribute between 50–75% of energy intake, but two-thirds of the vitamins and minerals naturally present in the unrefined staple are removed by the milling refinement process[42]. This fact, together with the wheat consumption observed in this study, amplifies justification for wheat flour as a potent fortification vehicle and why mandatory fortification is essential in this setting.

Plasma folate levels improved remarkably by month six of the study follow up, consistent with the rollout and scale-up of national food fortification program. These findings are consistent with other studies, that assessed the impact of the folic acid fortification programs[30,43–45], where significant increases in folate levels and declines in folate deficiency among women have been reported. A decline in the incidence of neural tube defects in Tanzania can be anticipated based on the decreased prevalence of folate deficiency observed, and in line with a 46% decline in NTD prevalence internationally[25]. Improving folate status may have other critical benefits. These may include reduced a risk of cardiovascular diseases[46], a reduced prevalence of anaemia[47,48], protection against other birth defects[49,50], and against allergies and hyper allergenic responses[51], and improved pregnancy outcomes[3–5] as well as overall healthier aging[52].

Despite above-mentioned benefits, several countries have not yet mandated fortification due to concerns for potential adverse effects from large-scale population based folic acid fortification[53]. Although the evidence is limited, these concerns include potential masking of vitamin B12 deficiency[54] and presence of unmetabolized folic acid in serum[55], although this has shown to be less likely in countries where increase in folic acid consumption does not exceed 400 μg/d [43]. Supraphysiological, and possibly even physiological, folate status may potentially favour malaria parasite growth and inhibit parasite clearance effect for some antimalarial drugs, hence increasing malaria recrudescence[56].Further risk-benefit analysis for folic acid fortification assessing secondary outcomes of interest such as the masking of B12 deficiency, increased levels of unmetabolized folic acid, and supraphysiologic folate status is warranted and important for future research. It is important noticing that about 50% of women were still deficient at 12 months, suggesting that continued efforts to enhance supplementation would be complementary[57].

Our study has several limitations. First, we did not collect and test food samples to assess the amount of fortificant contained. This limits our ability to correlate the rise in plasma folate levels with the levels of the fortificant in the foods. We therefore assumed that most of the wheat-based foods consumed by the participants following the launch of fortification were made from fortified flour. The slight reduction in the mean change in folate levels we observed at month 12 compared to month 6 was not statistically significant, and may have been due to chance. A Tanzania Food and Drug Authority (TFDA) post marketing surveillance report of April 2015 implies unsatisfactory and inconsistent levels of key micronutrients added in wheat flour and edible oil and fats[58]. Inadequate quality control at the mills, use of unacceptable packaging materials and improper handling and storage of the fortified products along the food chain are among the possible listed contributory factors[58]. Results from the 2015 Tanzania national fortification assessment coverage tool (FACT) cross-sectional survey indicates great variations in the fortification quality compared to Tanzania national standards[59]. Nationally only 18.9% of wheat flour and 3.3% of maize flour samples were adequately fortified, meanwhile similar household consumption patterns in rural and urban settings being reported for wheat flour and maize flour at 33.1% and 2.5% respectively[59]. Second, we experienced a gradual loss of participants over a period of 12 months of follow-up, with 33% lost to follow up at 12 months. There was however no difference in the baseline characteristics of participants’ loss to follow-up compared to the others in the study. When we restricted analysis to participants with complete follow-up at six and twelve months, a stronger protective association was seen at six months, with no significant association at twelve months. Epidemiological studies potentially suggest less risk of bias in cohort studies with up to 60% loss to follow-up or misses when such occurs at random[60]. The pre and post comparison design for folate levels in this cohort also reduces chances for bias. Due to full coverage nature of the fortification program, we chose a non-experimental design ‘before after’ design. In this case, causal attribution is not possible. However, the observed change is significantly large to have occurred due to other secular trends. Third, the study was conducted in an urban/ peri-urban setting where access to fortified foods may be different compared to rural parts of the country. In sub-Saharan Africa, wheat consumption is higher in urban compared to rural settings[61]. Therefore, maize flour (also under the fortification program but slower to start up in Tanzania) is intended to reach this rural population, however the number and nature of the maize industries in the landscape mean that quality control and therefore potential effectiveness may be more limited. Further understanding of access and consumption patterns, particularly in rural settings, remains critical for successful implementation of the National Food Fortification program in Tanzania. Hence, impact in rural areas should be a key objective for future studies. Finally, for logistics and quality reasons, we performed plasma instead of red blood cell folate assays. Red blood cell folate assays are recommended over plasma or serum folate assays, as they provide a measure of long term rather than recent intake for folic acid[15]. Competitive assay with folate binding protein used to measure folate concentration generally gives lower values compared to microbiological assay using *Lactobacillus rhamnosus*, currently recommended as the most reliable assay yielding comparable results for folate concentration across countries[15,34]. Although, it is possible that assays performed in separate baseline, 6 and 12 months’ batches introduce bias; it is implausible for this batch effect to explain the large effect size observed.

While improving folate status has many purported health benefits, when countries state a reason for including folic acid in flour fortification, it is usually to reduce the risk of neural tube defects (NTDs). The WHO new guidelines on optimal serum and red blood cell folate concentration in women of reproductive age for prevention of neural tube defects emphasizes on the importance of assessing “folate insufficiency”, instead of folate deficiency[34]. Folate insufficiency implies to levels below which women are at an increased risk of having an NTD affected pregnancy whereas folate deficiency levels forms a subset. This threshold has been recommended for blood folate assays and not for serum or plasma levels. The blood folate cutoff for folate insufficiency is higher than the cutoff point for folate deficiency[34]. This means that women who are not folate deficient, can be folate insufficient and at risk of having a baby with an NTD. Certainly the percent of women with folate deficiency represent the low end of the percent of women at risk of having an NTD-affected pregnancy; a more accurate representation of this risk is the percent of women with folate deficiency and insufficiency. To make predictions on NTDs at population level using plasma folate assays, relationship between both plasma and red blood cell folate needs first to be established in this setting. And therefore establish corresponding threshold for plasma folate assays using red blood cell folate levels.

## Conclusion

Similar to other countries with mandatory fortification programs, the wheat flour fortification program in Tanzania increased folate levels for urban women of reproductive age. Using the impact pathway adopted for this evaluation, we can make a plausible argument that a large scale wheat flour folic acid fortification program benefits women of reproductive age and hence having public health impact in Tanzania. Our findings are indicative of effectiveness of a large-scale food fortification program, and hence possible positive biological response to other fortificants like iron, zinc and B12. The net economic benefit of food fortification program in Tanzania is considerable; where $1 invested in central food fortification can result in an economic return of $8.22 or an increase in GDP of 0.58%. In addition, it is estimated that almost 6,800 deaths per year would be averted[10]. We recommend scale up of all selected food vehicles alike, especially maize flour fortification in settings where folate and other deficiencies are high.

## Supporting information

S1 File American Society for Nutrition license terms and conditions. Copyright approval by the American Society for Nutrition.
(PDF)

S2 File Folate data file. (CSV)
