# Supplementary material for: Large –scale wheat flour folic acid fortification program increases plasma folate levels among women of reproductive age in urban Tanzania
Source: PLoS One. 2017 Aug 10;12(8):e0182099. doi: 10.1371/journal.pone.0182099 (PMC5552223; doi:10.1371/journal.pone.0182099)
Supplement: S1 File — Copyright approval by the American Society for Nutrition. (PDF) [file pone.0182099.s001.pdf]

AMERICAN SOCIETY FOR NUTRITION LICENSE
TERMS AND CONDITIONS

Feb 27, 2017

This Agreement between Ramadhani A Noor ("You") and American Society for Nutrition ("American Society for Nutrition") consists of your license details and the terms and conditions provided by American Society for Nutrition and Copyright Clearance Center.

License Number	4040580923879
License date	
Licensed Content Publisher	American Society for Nutrition
Licensed Content Publication	The American Journal of Clinical Nutrition
Licensed Content Title	Effectiveness evaluation of the food fortification program of Costa Rica: impact on anemia prevalence and hemoglobin concentrations in women and children
Licensed Content Author	Reynaldo Martorell, Melany Ascencio, Luis Tacsan, Thelma Alfaro, Melissa F Young, O Yaw Addo, Omar Dary, Rafael Flores-Ayala
Licensed Content Date	Jan 1, 2015
Licensed Content Volume	101
Licensed Content Issue	1
Type of Use	Scholarly Journal
Requestor type	Academic Institution
Format	Print and electronic
Portion	Figures/table/illustration
Number of Figures/table/illustration	1
List of figures/table/ illustration	Figure 2: Program Impact Pathway for mass Fortification programs
Will you be translating?	No
Territory of distribution	Worldwide
Order reference number	
Title of new article	Large –scale wheat flour folic acid fortification program increases serum folate levels among women of reproductive age in urban Tanzania.
Publication the new article is in	Plos One
Publisher of new article	Plos One
Author of new article	Ramadhani Abdallah Noor
Expected publication date of new article	Mar 2017
Estimated size of new article (pages)	20
Requestor Location	Ramadhani A Noor<br>Africa Academy for Public Health (AAPH)<br>Box 79810<br>plot 802 Mwai Kibaki Rd

Dar Es Salaam, 79810
Tanzania, United Republic Of
Attn: Ramadhani A Noor

Billing Type	Invoice
Billing Address	Ramadhani A Noor<br>Africa Academy for Public Health (AAPH)<br>Box 79810<br>plot 802 Mwai Kibaki Rd<br>Dar Es Salaam, Tanzania, United Republic Of 79810<br>Attn: Ramadhani A Noor
Total	0.00 USD

Terms and Conditions

Terms and Conditions for RightsLink Licenses

1) This license agreement ("Agreement") is between the Customer as identified by the user in **RightsLink** ("Customer") and The American Society for Nutrition, Inc, a nonprofit corporation with offices at 9211 Corporate Blvd., Suite 300, Rockville, MD 20850 USA ("ASN"), regarding content published in one of the following ASN journals: *The American Journal of Clinical Nutrition (AJCN)* or *The Journal of Nutrition (JN) or Advances in Nutrition*. The license administrator for this Agreement is the Copyright Clearance Center (CCC).

2) Introduction

The publisher of this copyrighted material is ASN. By clicking "accept" in connection with completing this licensing transaction, you agree that the following terms and conditions apply to this transaction (along with the Billing and Payment terms and conditions established by CCC, at the time that you opened your **RightsLink** account and that are available at any time at http://myaccount.copyright.com.

3) ASN occasionally publishes material for which it does not hold copyright; in such instances, ASN identifies and properly cites the appropriate copyright holder. It is the Customer's responsibility to verify that ASN holds copyright for which the Customer is requesting permission. If the material is credited to another source, then ASN does not have the authority to grant permission for its reuse and this license is to be considered invalid.

4) Limited License

ASN hereby grants to you a non-exclusive license to use this material. Licenses are for one-time use only with a maximum distribution equal to the number that you identified in the licensing process. Except when material is licensed for reuse in a scholarly journal, magazine, newsletter, newspaper, book, or textbook, in which case permission extends to reprints of the product produced for publisher restocking, any form of republication must be completed within one year from the date hereof (although copies prepared before then may be distributed thereafter). Reprints must not be new products nor marketed as such: **reuse in new volumes or new editions must be licensed explicitly**. Any form of republication must be completed within one year from the date hereof (although copies prepared before then may be distributed thereafter); and any electronic posting is limited to the time specified in this request, if any. ASN asks that commercial organizations limit distribution of photocopies to within the organization requesting permission. Requests to distribute content copyrighted by ASN outside of the requesting organization should be processed as reprints or ePrints. Use of more than 50% of an article copyrighted by ASN in a single new work must be reviewed separately by ASN staff.

5) Geographic Rights

Licenses may be exercised solely in the country or countries specified by the Customer on the Quick Price or Additional Data page in **RightsLink**. However, in cases in which ASN has exclusive republication licensees in specific countries, licenses to reproduce full-text material in these countries may be revoked by ASN. In such cases, the Customer would be

referred to the license agent in the respective country or countries. For questions about whether this would apply to your order, please contact ASN at publications@nutrition.org.

6) Alterations/Modifications

Except for adaptations of tables and figures and for translations of content as described below, material may not be modified. Translations of figures, tables, and excerpts are permitted as transactions within **RightsLink**. Translations of full-text materials must be approved by ASN before the Customer can proceed with the **RightsLink** transaction. Full-text translation requests processed through CCC will be considered invalid unless the Customer enters the ASN-issued approval code for the translation. A copy of all full-text translations must be sent to ASN.

7) Reservation of Rights

ASN reserves all rights not specifically granted in the combination of (i) the license details provided by you and accepted in the course of this licensing transaction, (ii) these terms and conditions, and (iii) CCC's Billing and Payment terms and conditions.

8) License Contingent on Payment

While you may exercise the rights licensed immediately upon issuance of the license at the end of the licensing process for the transaction, provided that you have disclosed complete and accurate details of your proposed use, no license is finally effective unless and until full payment is received from you (either by ASN or by CCC) as provided in CCC's Billing and Payment terms and conditions. If full payment is not received on a timely basis, then any license preliminarily granted shall be deemed automatically revoked and shall be void as if never granted. Further, in the event that you breach any of these terms and conditions or any of CCC's Billing and Payment terms and conditions, the license is automatically revoked and shall be void as if never granted. Use of materials as described in a revoked license, as well as any use of the materials beyond the scope of an unrevoked license, may constitute copyright infringement and publisher reserves the right to take any and all action to protect its copyright in the materials.

9) Copyright Notice: Disclaimer

You must give complete credit to the original source in connection with any reproduction or adaptation of the licensed material: 'Am J Clin Nutr (year;volume:page range), American Society for Nutrition,' or 'J Nutr (year;volume:page range), American Society for Nutrition;.'Adv Nutr (year;volume:page range), American Society for Nutrition.'For translations: Figures or tables cannot be used in advertisements.

Full-text translations of articles published in *The Journal of Nutrition* must include the following disclaimer: *Translated from the original into LANGUAGE by TRANSLATOR'S NAME. The translator assumes responsibility for the accuracy of the translation. The American Society for Nutrition is not responsible for translation errors. Readers are encouraged to access the original publication at http://jn.nutrition.org.*For photocopies and in-house reprints:

The article will be reprinted in its entirety without change.

No material will be attached to the reprints, and the reprint will be used for educational purposes only, not to promote, sell, or in any way endorse a product.

The citation appearing on the first page of the article must appear in the reprints.

As a courtesy, please notify the author of your intended reuse of his or her content.

All reprints must have reprinted on each:*The American Society for Nutrition, Inc., does not endorse any commercial enterprise.*

10) Warranties

ASN makes no representations or warranties with respect to the licensed material.

11) Indemnity

You hereby indemnify and agree to hold harmless ASN and CCC, and their respective officers, directors, employees, and agents, from and against any and all claims arising out of your use of the licensed material other than as specifically authorized pursuant to this license.

12) No Transfer of License
This license is personal to you, but may be assigned or transferred by you to a business associate (or to your employer) if you give prompt written notice of the assignment or transfer to ASN. No such assignment or transfer shall relieve you of the obligation to pay the designated license fee on a timely basis (although payment by the identified assignee can fulfill your obligation).
13) No Amendment Except in Writing
This license may not be amended except in a writing signed by both parties (or, in the case of ASN, by CCC on ASN's behalf).
14) Objection to Contrary Terms
ASN hereby objects to any terms contained in any purchase order, acknowledgment, check endorsement or other writing prepared by you, which terms are inconsistent with these terms and conditions or CCC's Billing and Payment terms and conditions. These terms and conditions, together with CCC's Billing and Payment terms and conditions (which are incorporated herein), comprise the entire agreement between you and ASN (and CCC) concerning this licensing transaction. In the event of any conflict between your obligations established by these terms and conditions and those established by CCC's Billing and Payment terms and conditions, these terms and conditions shall control.
15) Jurisdiction
This license Agreement contains the entire understanding of the parties with respect to the licensed content and can be modified only by a signed, written agreement. This Agreement shall be construed in accordance with the laws of the State of Maryland and the US copyright laws. If any term of this Agreement shall be found invalid by any court of competent jurisdiction, such provision shall be enforced to the fullest extent that it is valid and enforceable under applicable law, and all other provisions of this Agreement shall remain in full force and effect. For state and local governments, the Terms and Conditions do not apply when in conflict with existing statues.
16) Other
There are additional terms and conditions, established by Copyright Clearance Center, Inc. ("CCC") as the administrator of this licensing service that relate to billing and payment for licenses provided through this service. Those terms and conditions apply to each transaction as if they were restated here. As a user of this service, you agreed to those terms and conditions at the time that you established your account, and you may see them again at any time at http://myaccount.copyright.com.
Terms and Conditions for Content Services

Subject to these terms of use, any terms set forth on the particular order, and payment of the applicable fee, you may make the following uses of the ordered materials:

Content Rental: You may access and view a single electronic copy of the materials ordered for the time period designated at the time the order is placed. Access to the materials will be provided through a dedicated content viewer or other portal, and access will be discontinued upon expiration of the designated time period. An order for Content Rental does not include any rights to print, download, save, create additional copies, to distribute or to reuse in any way the full text or parts of the materials.

Content Purchase: You may access and download a single electronic copy of the materials ordered. Copies will be provided by email or by such other means as publisher may make available from time to time. An order for Content Purchase does not include any rights to create additional copies or to distribute copies of the materials.

The materials may be accessed and used only by the person who placed the Order or the person on whose behalf the order was placed and only in accordance with the terms included in the particular order.

Special Terms: <%=specialTerms%>
v 2.7

Questions? customercare@copyright.com or +1-855-239-3415 (toll free in the US) or +1-978-646-2777.
